# Supplementary material for: Discovery of Selective SIRT2 Inhibitors as Therapeutic Agents in B-Cell Lymphoma and Other Malignancies
Source: Molecules. 2020 Jan 21;25(3):455. doi: 10.3390/molecules25030455 (PMC7036909; doi:10.3390/molecules25030455)
Supplement: Supplementary file 1 [file molecules-25-00455-s001.pdf]

Supporting Information

Discovery of isoform selective SIRT2 inhibitors as therapeutic agents in B-cell lymphoma and other malignancies

Sarwat Chowdhury,[1] Angela Park,[1] Uyen Lao,[1] Joanne H. Hsu,[1] Smitha Sripathy,[1] Tyler Loe,[1] Antonio Bedalov[1] and Julian A. Simon[1,2*]

AUTHOR ADDRESS [1]Clinical Research Division, Fred Hutchinson Cancer Research Center, Seattle, WA USA

[2]Human Biology and Public Health Sciences Divisions, Fred Hutchinson Cancer Research Center, Seattle, WA USA

Table of Contents

General Chemistry 3

Representative Procedure (I) for Preparation of Substituted 2-Hydroxy-1-Naphthalene Carbaldehydes (**7**) 3

Preparation of ethyl 3-(4-acetamidophenyl)-3-oxopropanoate (**11**) 5

Representative Procedure (II) for Preparation of Substituted 2-Benzoyl-3H-benzo-[f]chromen-3-ones (**13**) 5

Representative Procedure (III) for Preparation of Substituted 2-(benzoyl)-1H,2H-naphtho[2,1-b]pyran-3-one (**14**) 12

General Chemistry. Thin layer chromatography was carried out using Merck 60 F254 silica gel plates using appropriate solvent mixtures. Solvents were ACS reagent grade and anhydrous solvents (Aldrich and Acros) were used as received. Medium pressure chromatography was carried out using Biotage Isolera with Silicycle HP cartridges for compound purification. LCMS was performed using an Agilent 1100 HPLC system equipped with a Waters XTerra MS C18 5 µm, 4.6 Å~ 50 mm column or Poroshell 120 EC-C18 4.6x100 mm column using an Agilent photodiode array detector and an in-line Agilent 6130 single quadrupole mass spectrometer. Analytical HPLC method involved gradient elution from 0 to 95% acetonitrile in water (0.1% formic acid) over 6 min. Agilent ChemStation software was used to develop methods. Final purity of the compounds was determined by ^{1}H-NMR or by analyzing chromatogram of the products at 210, 254 and 280 nm. All compounds reported were >95% pure as judged by HPLC.

Representative Procedure (I) for Preparation of Substituted 2-Hydroxy-1-Naphthalene Carbaldehydes (7).

6-Bromo-2-hydroxynaphthalene-1-carbaldehyde (7a).

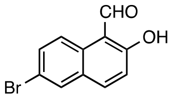

A solution of $TiCl_4$ (1.0 M in CH_2Cl_2, 27 mmol, 27 mL) in dichloromethane was treated with a solution of dichloromethyl methyl ether (13.5 mmol) in 1,2-dichloroethane (3 ml) at 0 °C for 15 min and the mixture was allowed to stir for 30 min. A solution of 6-bromo-2-hydroxy naphthalene (13.5 mmol) in CH_2Cl_2 (30 mL) was next added dropwise, and the reaction was stirred overnight with gradual warm up to the room temperature. The reaction was quenched by adding 1 N HCl (10 mL). The aqueous layer was extracted with CH_2Cl_2 (x3) and the organic layers were then combined, dried over Na_2SO_4, and reduced to dryness to afford a reddish-brown residue which was further purified using medium pressure chromatography using a gradient EtOAc/hexane solvent system (1−10% EtOAc) to yield 1.4 g (1.20 g, 5.8 mmol, 43%) of the desired product as a white powder.

^{1}H NMR (300 MHz, DMSOd$_6$) δ 12.01-11.79 (bs, 1H), 10.76 (s,1H), 8.94 (d, *J = 9.0 Hz*, 1H), 8.17 (d, *J = 1.5 Hz*, 1H), 8.11 (d, *J = 9.0 Hz*, 1H), 7.73 (dd, *J = 9.0, 1.8 Hz*, 1H), 7.30 (d, *J = 9.0 Hz*, 1H). LRMS: m/z = 248.9 (M−H)$^-$.

2-Hydroxy-6-methoxynaphthalene-1-carbaldehyde (7b)

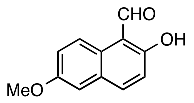

0.68 g, 3.36 mmol, 37%. ^{1}H NMR (300 MHz, DMSOd$_6$) δ 10.73 (s, 1H), 9.71 (s,1H), 8.96 (d, *J = 9.3 Hz,* 1H), 8.08 (d, *J = 9.3 Hz,* 1H), 7.50 (d, *J = 9.0 Hz,* 1H), 7.20 (dd, *J = 9.3, 2.4 Hz,* 1H), 7.16 (s, 1H), 4.01 (s, 3H). LRMS: m/z = 203.1 $(M+H)^+$.

3-Bromo-2-hydroxynaphthalene-1-carbaldehyde (7c). Described earlier.[1]

7-Bromo-2-hydroxynaphthalene-1-carbaldehyde (7d). Described earlier. [1]

2-Hydroxy-6-methylnaphthalene-1-carbaldehyde (7e).

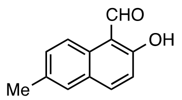

92.2 mg, 78%. ^{1}H NMR (300 MHz, DMSOd$_6$) δ 11.87 (s, 1H), 10.79 (s, 1H), 8.82 (d, *J = 8.7 Hz*, 1H), 8.04 (d, *J = 9.0 Hz*, 1H), 7.66 (s, 1H), 7.46 (d, *J = 8.4 Hz*, 1H), 7.20 (d, *J = 9.0 Hz*, 1H), 2.43 (s, 3H). LRMS m/z = 187.1 $(M+H)^+$.

Preparation of 2-chloro-6-hydroxy-quinoline carbaldehyde (9).

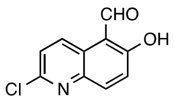

2(1H)-quinolinone (966.9 mg, 6.0 mmol) was taken up in trifluoroacetic acid (8 mL). To this solution was added hexamethylenetetramine (2.0 equiv., 12.0 mmol, 1.682 g), and the resulting mixture was then heated at 100 °C for 3 h. The reaction was allowed to cool, methanol (120 mL) was added, and the solvent was evaporated to afford a dark residue. Water (15 mL) was added, and the resulting precipitate was filtered and dried to obtain 2-quinolone-5-carbaldehyde in 95% yield (1.05 g, 5068 mmol) as pale-yellow solid. LRMS m/z = 188.0 $(M-H)^+$.

The precipitate 189 mg (1.0 mmol) was dissolved in DMF (1.0 mL), the suspension cooled to 0 °C, whereupon phosphorus oxyhalide ($POCl_3$) (3.0 mmol, 0.283 ml) was added dropwise. The light gray colored suspension was then stirred at room temp overnight. Ice-cold water (4 mL) was added, and the precipitate was filtered and dried under a vacuum to yield the corresponding aldehyde as a pure product (193 mg, 0.93 mmol, 93%). ^{1}H NMR (300 MHz, DMSOd$_6$) δ 11.94 (s, 1H), 10.70 (s,1H), 9.39 (d, *J = 9.0 Hz*, 1H), 8.11 (d, *J = 9.3 Hz*, 1H), 7.68 (d, *J = 9.0 Hz*, 1H), 7.56 (d, *J = 9.3 Hz*, 1H). LRMS m/z = 208.0 $(M+H)^+$.

Preparation of ethyl 3-(4-acetamidophenyl)-3-oxopropanoate (11).

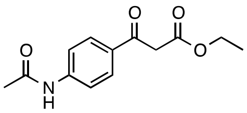

Ethyl (4-nitrophenyl)-3-oxopropanoate (273 mg, 1.16 mmol) was dissolved in 5 mL EtOH and subsequently treated with acetic acid (2 mL), HCl (2M, 1 mL) and 0.30 g iron power (-70 mesh). The mixture was refluxed for 2 h where upon the cooled down, filtered through Celite and concentrated under evacuo. The resultant solid was taken up in methylene chloride, washed with water (x2), dried over Na_2SO_4 and concentrated to afford ethyl 3-(4-aminophenyl)-3-oxopropanoate product which was forwarded to the next step without futher purification. LRMS m/z = 208.1 $(M+H)^+$.

Ethyl 3-(4-aminophenyl)-3-oxopropanoate (0.8 mmol) was dissolved in 4 mL of methylene chloride and treated with Et_3N (0.33 mL, 2.4 mmol) and neat acetyl chloride (1.0 mmol, 0.08 mL) and the solution was stirred at rt for 3 h. The reaction was quenched by addition of water and organic layer was separated. Aqueous layer was extracted with EtOAc (x2), combined organics dried over Na_2SO_4 and reduced to dryness to afford ethyl 3-(4-acetamidophenyl)-3-oxopropanoate (196mg, 99%). LRMS m/z = 250.0 $(M+H)^+$.

Representative Procedure (II) for Preparation of Substituted 2-Benzoyl-3H-benzo-[f]chromen-3-ones (13).

8-Bromo-2-(3-bromobenzoyl)-3H-benzo[f]chromen-3-one (13a)

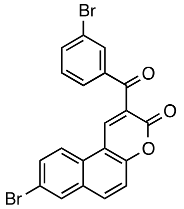

To a solution of substituted 6-bromo-2-hydroxyl naphthaldehyde (124 mg, 0.5 mmol) in ethanol (5 mL) was added the corresponding ethyl-(3-bromophenyl)-3-oxopropanoate 95.5 μL (0.5 mmol). Piperidine (5 drops) was next added, and the reaction was heated under reflux for 2 h whereupon the reaction was allowed to cool down. The yellowish precipitate obtained was collected by filtration and washed with ethanol several times to get the condensation product 8-Bromo-2-(3-bromobenzoyl)-3H-benzo[f]chromen-3-one, 200 mg (0.20 g, 0.44 mmol, 88%). This product was forwarded to the next step without further purification. ^{1}H NMR (300 MHz, DMSOd$_6$) δ 9.24 (s, 1H), 8.59 (d, *J = 9.0 Hz*, 1H), 8.43 (s, 1H), 8.31 (d, *J = 9.0 Hz*, 1H), 8.18 (s, 1H), 7.99 (d, *J = 7.8 Hz*, 1H), 7.90 (t, *J =7.5 Hz*, 2H), 7.74 (d, *J = 9.3 Hz*, 1H), 7.53 (t, *J = 7.8 Hz*, 1H). LRMS m/z = 457.0 (M+H)$^+$.

8-Bromo-2-(4-methylbenzoyl)-3H-benzo[f]chromen-3-one (13b)

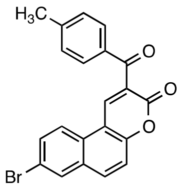

819 mg (2.09 mmol, 58%). ^{1}H NMR (300 MHz, DMSOd$_6$) δ 9.17 (s, 1H), 8.58 (d, *J = 9.0 Hz*, 1H), 8.46-8.38 (bs, 1H), 8.30 (d, *J = 9.0 Hz*, 1H), 7.95-7.81 (m, 3H), 7.73 (d, *J = 9.0 Hz*, 1H), 7.37 (d, *J = 7.8 Hz*, 2H), 2.41 (s, 3H). LRMS m/z = 393.0 (M+H)$^+$.

8-Bromo-2-(3-methylbenzoyl)-3H-benzo[f]chromen-3-one (13c)

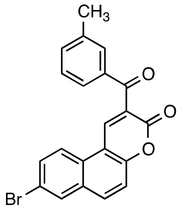

478 mg (1.50 mmol, 81%). ^{1}H NMR (300 MHz, DMSOd_6) δ 9.17 (s, 1H), 8.59 (d, *J = 9.3 Hz*, 1H), 8.42 (s, 1H), 8.30, (d, *J = 9.0 Hz*, 1H), 7.93-7.67 (m, 4H), 7.59-7.37 (m, 2H), 2.38 (s, 3H). LRMS $[ES]^+$: m/z = 393.0 $(M+H)^+$.

8-Bromo-2-(2-methylbenzoyl)-3H-benzo[f]chromen-3-one (13d)

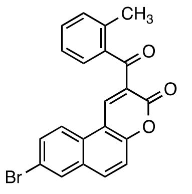

588 mg (1.50 mmol, 74%). 478 mg (1.50 mmol, 81%). ^{1}H NMR (300 MHz, DMSOd_6) δ 9.23 (s, 1H), 8.59 (d, *J = 9.3 Hz*, 1H), 8.42 (s, 1H), 8.32 (d, *J = 9.0 Hz*, 1H), 8.17 (bs, 1H), 7.99 (d, *J = 7.8 Hz*, 1H), 7.94-7.88 (m, 2H), 7.74 (d, *J = 9.3 Hz*, 1H), 7.53 (t, *J = 7.8 Hz*, 1H), 2.50 (s, 3H).

8-Bromo-2-benzoyl-3H-benzo[f]chromen-3-one (13e)

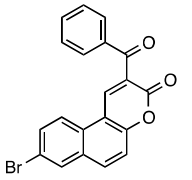

982 mg (2.6 mmol, 77%). ^{1}H NMR (300 MHz, DMSOd_6) δ 9.21 (s, 1H), 8.59 (d, *J = 9.3 Hz*, 1H), 8.42 (s, 1H), 8.31 (d, *J = 9.3 Hz*, 1H), 8.00 (d, *J = 7.8 Hz*, 2H), 7.87 (d, *J = 8.7 Hz*, 1H), 7.72 (t, *J 8.4 Hz*, 1H), 7.56 (t, *J = 7.8 Hz*, 1H).

8-Bromo-2-(4-chlorobenzoyl)-3H-benzo[f]chromen-3-one (13f)

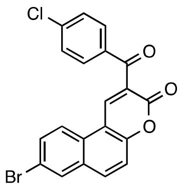

284 mg (0.69 mmol, 69%). ^{1}H NMR (300 MHz, DMSOd_6) δ 9.22 (s, 1H), 8.58 (d, *J = 9.0 Hz*, 1H), 8.42 (s, 1H), 8.31 (d, *J = 9.3 Hz*, 1H), 8.02 (d, *J = 8.7 Hz*, 2H), 7.87 (d, *J = 9.0 Hz*, 1H), 7.73 (d, *J = 9.3 Hz*, 1H), 7.63 (d, *J = 8.4 Hz*, 2H).

8-Bromo-2-(4-trifluoromethylbenzoyl)-3H-benzo[f]chromen-3-one **(13g)**

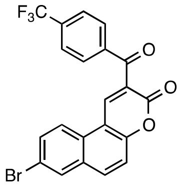

430 mg (0.97 mmol, 80%). ^{1}H NMR (300 MHz, DMSOd$_6$) δ 9.29 (s, 1H), 8.60 (d, *J = 9.0 Hz*, 1H), 8.42 (s, 1H), 8.33 (d, *J = 9.0 Hz*, 1H), 8.19 (d, *J = 9.0 Hz*, 2H), 7.99-7.84 (m, 3H), 7.74 (d, *J = 9.0 Hz*, 1H).

8-Bromo-2-(3-trifluoromethylbenzoyl)-3H-benzo[f]chromen-3-one (13h)

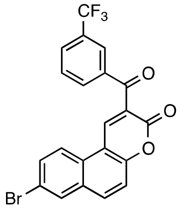

275 mg (0.62 mmol, 62%). ^{1}H NMR (300 MHz, DMSOd$_6$) δ 9.28 (s, 1H), 8.59 (d, *J = 9.0 Hz*, 1H), 8.42 (s, 1H), 8.37-8.24 (m, 3H), 8.07 (d, *J = 7.8 Hz*, 1H), 7.88 (d, *J = 9.0 Hz*, 1H), 7.85-7.69 (m, 2H).

8-Bromo-2-(4-bromobenzoyl)-3H-benzo[f]chromen-3-one (13i)

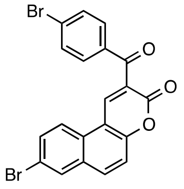

453 mg (0.99 mmol, 88%). ^{1}H NMR (300 MHz, DMSOd$_6$) δ 9.23 (s, 1H), 8.59 (d, *J = 9.3 Hz*, 1H), 8.42 (s, 1H), 8.31 (d, *J = 9.3 Hz*, 1H), 7.98-7.86 (m, 3H), 7.81-7.69 (m, 3H).

8-Bromo-2-(4-methoxybenzoyl)-3H-benzo[f]chromen-3-one (13j)

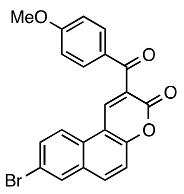

535 mg (1.31 mmol, 81%). ^{1}H NMR (300 MHz, DMSOd$_6$) δ 9.14 (s, 1H), 8.59 (d, *J = 9.0 Hz*, 1H), 8.41 (s, 1H), 8.29 (d, *J = 9.3 Hz*, 1H), 7.98 (d, *J = 9.3 Hz*, 2H), 7.86 (d, *J = 9.0 Hz*, 1H), 7.73 (d, *J = 8.7 Hz*, 1H), 7.08 (d, *J = 7.8 Hz*, 2H), 3.88 (s, 3H).

8-Bromo-2-(4-fluorobenzoyl)-3H-benzo[f]chromen-3-one (13k)

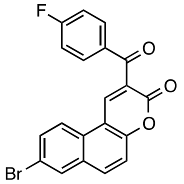

400 mg (1.01 mmol, 67%). ^{1}H NMR (300 MHz, DMSOd$_6$) δ 9.21 (s, 1H), 8.58 (d, *J = 9.3 Hz*, 1H), 8.41 (s, 1H), 8.30 (d, *J = 9.3 Hz*, 1H), 8.10 (dd, *J = 7.8, 2.1 Hz*, 2H), 7.87 (d, *J = 9.0 Hz*, 1H), 7.73 (d, *J = 9.0 Hz*, 1H), 7.39 (t, *J = 8.7 Hz*, 2H). LRMS [ES]$^+$: m/z = 397.0 (M+H)$^+$.

8-bromo-2-(pyridine-4-carbonyl)benzo[f]chromen-3-one (13l)

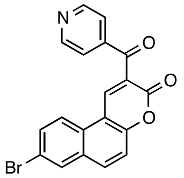

90 mg (0.24 mmol, 16%). ^{1}H NMR (300 MHz, DMSOd$_6$) δ 9.31 (s, 1H), 8.83 (dd, *J = 6.3, 1.8 Hz*, 2H), 8.61 (d, *J = 9.0 Hz*, 1H), 8.43 (d, *J = 2.1 Hz*, 1H), 8.34 (d, *J = 9.0 Hz*, 1H), 7.93-7.82 (m, 3H), 7.75 (d, *J = 9.0 Hz*, 1H). LRMS [ES]$^+$: m/z = 380.7 (M+H)$^+$.

8-bromo-2-(pyridine-3-carbonyl)benzo[f]chromen-3-one (13m)

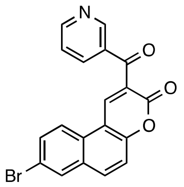

230 mg (0.60 mmol, 44%). ^{1}H NMR (300 MHz, DMSOd$_6$) δ 9.29 (s, 1H), 9.12 (s, 1H), 8.84 (d, *J = 4.8 Hz*, 1H), 8.60 (d, *J = 9.0 Hz*, 1H), 8.42 (s, 1H), 8.39-8.28 (m, 2H), 7.88 (d, *J = 9.0 Hz*, 1H), 7.74 (d, *J = 9.0 Hz*, 1H), 7.60 (dd, *J= 7.8, 4.8 Hz*, 1H). LRMS [ES]$^+$: m/z = 380.0 (M+H)$^+$.

***N*-(4-{8-bromo-3-oxobenzo[f]chromene-2-carbonyl}phenyl)acetamide (13n)**

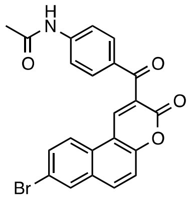

114 mg (0.26 mmol, 33%). LRMS: m/z 436.0 (M+H)$^+$.

2-(3-bromobenzoyl)-3H-benzo[f]chromen-3-one (13o)

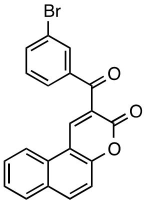

0.78 mg (2.06 mmol, 89%). ^{1}H NMR (300 MHz, DMSOd$_6$) δ 9.25 (s, 1H), 8.61 (d, *J = 8.4 Hz*, 1H), 8.34 (d, *J = 9.0 Hz*, 1H), 8.17 (bt, *J = 1.5 Hz*, 1H), 8.11 (*J = 7.8 Hz*, 1H), 7.98 (d, *J = 7.8 Hz*, 1H), 7.90 (td, *J = 8.1, 0.9 Hz*, 1H), 7.77 (t, *J= 8.1 Hz*, 1H), 7.72-7.61 (m, 2H), 7.52 (t, *J = 8.1Hz*, 1H). LRMS [ES]$^+$: m/z = 379.0 (M+H)$^+$.

8-methoxy 2-(3-bromobenzoyl)-3H-benzo[f]chromen-3-one (13p)

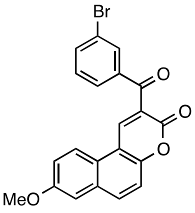

87.5 mg (0.21 mmol, 22%). ^{1}H NMR (300 MHz, DMSOd$_6$) δ 9.20 (s, 1H), 8.53 (d, *J = 9.6 Hz*, 1H), 8.24 (d, *J = 9.0 Hz*, 1H), 8.15 (s, 1H), 7.97(*J = 7.8 Hz*, 1H), 7.90 (d, *J = 7.8 Hz*, 1H), 7.64

(d, *J = 9.3 Hz*, 1H), 7.57 (d, *J= 2.7 Hz*, 1H), 7.52 (t, *J = 7.8 Hz*, 1H), 7.40 (dd, *J = 9.3, 2.7 Hz*, 1H), 3.92 (s, 3H). LRMS $[ES]^+$: m/z = 409.8 $(M+H)^+$.

5-bromo-2-(3-bromobenzoyl)benzo[f]chromen-3-one (13q)

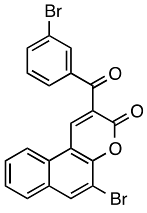

376 mg (0.82 mmol, 98%). ^1H NMR (300 MHz, $DMSOd_6$) δ 9.26 (s, 1H), 8.76 (s, 1H), 8.62 (d, *J = 8.4 Hz*, 1H), 8.22 (t, *J = 1.8 Hz*, 1H), 8.10 (d, *J = 8.7 Hz*, 1H), 8.02 (d, *J = 8.1 Hz*, 1H), 7.92 (d, *J = 7.8 Hz*, 1H), 7.79 (t, *J = 7.8 Hz*, 1H), 7.73-7.66 (m, 1H). 7.53 (t, *J = 7.8 Hz*, 1H). LRMS $[ES]^+$: m/z = 456.9 $(M+H)^+$.

9-bromo-2-(3-bromobenzoyl)benzo[f]chromen-3-one (13r)

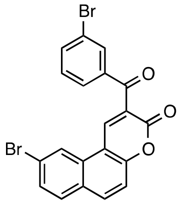

393 mg (0.86 mmol, 90%). ^1H NMR (300 MHz, $DMSOd_6$) δ 9.31 (s, 1H), 8.92 (d, *J = 1.2 Hz*, 1H), 8.34 (d, *J = 9.0 Hz*, 1H), 8.18 (t, *J = 1.8 Hz*, 1H), 8.07 (d, *J = 8.7 Hz*, 1H), 8.00 (d, *J = 7.8 Hz*, 1H), 7.91 (d, *J = 7.8 Hz*, 1H), 7.79 (dd, *J= 8.7, 1.8 Hz*, 1H), 7.71 (d, *J = 9.0 Hz*, 1H), 7.53 (t, *J = 7.8 Hz*, 1H). LRMS $[ES]^+$: m/z = 356.9 $(M+H)^+$.

8-methyl-2-(4-trifluoromethylbenzoyl)benzo[f]chromen-3-one (13s)

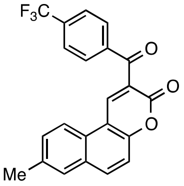

362 mg (0.94 mmol, 67%). ^{1}H NMR (300 MHz, DMSOd$_6$) δ 9.28 (s, 1H), 8.53 (d, *J = 8.7 Hz*, 1H), 8.25 (d, *J = 9.0 Hz*, 1H), 8.17 (d, *J = 8.1 Hz*, 2H), 7.96-7.87 (m, 3H), 7.67-7.58 (m, 2H), 7.52 (s, 3H).

Representative Procedure (III) for Preparation of Substituted 2-(benzoyl)-1H,2H-naphtho[2,1-b]pyran-3-one (14).

8-bromo-2-(3-bromobenzoyl)-1H,2H-naphtho[2,1-b]pyran-3-one (14a)

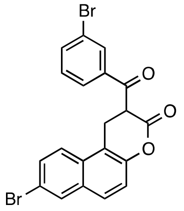

Benzoyl coumarin **13a** (0.20 g, 0.44 mmol) was dissolved in dry pyridine (2 mL). To this solution was added $NaBH_4$ (1.25 equiv., 0.55 mmol, 20.9 mg), and the reaction was stirred at room temp for 3 h. The mixture was then poured in cold 2 M hydrochloric acid (5 mL), which resulted in a white precipitate. The precipitate was washed several times with water, dried under a vacuum to yield the corresponding 2-benzoyl-1,2-dihydrocoumarin as white powder (0.17 g, 0.36mmol, 82%) which was taken to the next step without purification. ^{1}H NMR (300 MHz, DMSOd$_6$) δ 8.29 (d, *J = 7.8 Hz*, 2H), 8.09 (d, *J = 7.8 Hz*, 1H), 8.03-7.87 (m, 3H), 7.71 (d, *J = 9.9 Hz*, 1H), 7.53 (t, *J = 7.8 Hz*, 1H), 7.42 (d, *J = 9.0 Hz*, 1H), 5.38 (dd, *J= 11.4, 6.6 Hz*, 1H), 3.57 (dd, *J = 16.8, 11.7, 6.6 Hz*, 2H). LRMS 458.9 $(M+H)^+$.

8-bromo-2-(4-methylbenzoyl)-1H,2H-naphtho[2,1-b]pyran-3-one (9)

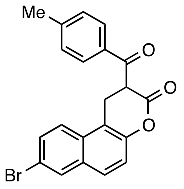

208 mg (0.53 mmol, 88%). ^{1}H NMR (300 MHz, DMSOd$_6$) δ 8.26 (d, *J = 2.1 Hz*, 1H), 8.04-7.89 (m, 4H), 7.68 (dd, *J = 9.0, 2.1 Hz*, 1H), 7.44-7.30 (m, 3H), 5.32 (dd, *J= 9.9, 6.6 Hz*, 1H), 3.67 (ddd, *J= 16.8, 9.9, 6.6Hz*, 2H), 2.40 (s, 3H).

8-bromo-2-(3-methylbenzoyl)-1H,2H-naphtho[2,1-b]pyran-3-one (14b)

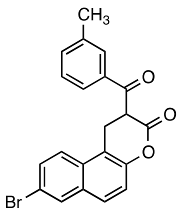

336 mg (0.85 mmol, 95%). ^{1}H NMR (300 MHz, DMSOd$_6$) δ 8.27(d, *J = 1.8 Hz*, 1H), 8.02-7.87 (m, 4H), 7.69 (dd, *J = 9.0, 1.8 Hz*, 1H), 7.55-7.39 (m, 3H), 5.35 (dd, *J= 10.5, 7.2 Hz*, 1H), 3.68 (ddd, *J = 16.2, 10.5, 7.5Hz*, 2H), 2.38 (s, 3H). LRMS [ES]$^+$: m/z = 395.9 (M + H)$^+$.

8-bromo-2-(2-methylbenzoyl)-1H,2H-naphtho[2,1-b]pyran-3-one (14c)

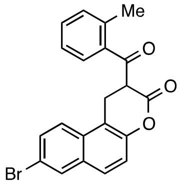

267 mg (0.68 mmol, 81%). LRMS [ES]$^+$: m/z = 395.8 (M - H)$^+$.

8-bromo-2-(benzoyl)-1H,2H-naphtho[2,1-b]pyran-3-one (14d)

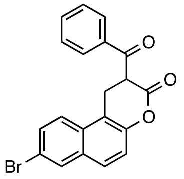

760 mg (2.00 mmol, 95%). ^{1}H NMR (300 MHz, DMSOd$_6$) δ 8.27(d, *J = 2.1 Hz*, 1H), 8.10 (d, *J =7.5 Hz*, 2H), 7.95 (t, *J = 9.3,* 2H), 7.75-7.66 (m, 2H), 7.56 (t, *J= 7.8 Hz*, 2H), 7.42 (d, *J= 8.7 Hz*, 1H), 5.37 (dd, *J= 10.5, 6.9 Hz*, 1H), 3.69 (ddd, *J = 15.9, 10.5, 6.9 Hz*, 2H).

8-Bromo-2-(4-chlorobenzoyl)-1H,2H-naphtho[2,1-b]pyran-3-one (14e)

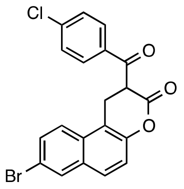

287 mg (5.96 mmol, 99%). ^{1}H NMR (300 MHz, DMSOd$_6$) δ 8.27 (d, *J = 1.8 Hz*, 1H), 8.12 (d, *J = 8.7 Hz*, 2H), 7.96 (dd, *J = 11.4, 9.6 Hz*, 2H), 7.70 (dd, *J = 8.7, 2.1 Hz*, 1H), 7.64 (d, *J = 8.4 Hz*, 2H), 7.22 (d, *J = 9.0,* 1H), 5.35 (dd, *J= 11.1, 6.9 Hz*, 1H), 3.78 (ddd, *J = 16.3, 11.1, 6.9 Hz*, 2H).

8-Bromo-2-(4-trifluoromethylbenzoyl)-1H,2H-naphtho[2,1-b]pyran-3-one (14f)

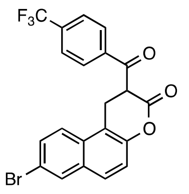

266 mg (0.60 mmol, 88%). ^{1}H NMR (300 MHz, DMSOd$_6$) δ 8.39-8.24 (m, 3H), 8.07-7.90 (m, 4H), 7.71 (dd, *J = 9.0, 2.1 Hz*, 1H), 7.43 (d, *J = 9.0 Hz,* 1H), 5.43 (dd, *J= 11.4, 6.9 Hz*, 1H), 3.68 (ddd, *J = 16.8, 11.4, 6.9 Hz*, 2H).

8-Bromo-2-(3-trifluoromethylbenzoyl)-1H,2H-naphtho[2,1-b]pyran-3-one (14g)

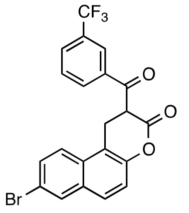

233 mg (0.52 mmol, 92%). ^{1}H NMR (300 MHz, DMSOd$_6$) δ 8.45 (s, 1H), 8.40 (d, *J = 7.8 Hz*, 1H), 8.29 (d, *J = 2.1 Hz*, 1H), 8.10 (d, *J = 7.5 Hz*, 1H), 7.95 (dd, *J = 12.6, 9.0 Hz*, 2H), 7.82 (t, *J = 7.8 Hz*, 1H), 7.72 (dd, *J = 9.0, 1.8 Hz*, 1H), 7.44 (d, *J = 9.0,* 1H), 5.48 (dd, *J= 11.7, 6.9 Hz*, 1H), 3.69 (ddd, *J = 16.5, 11.7, 6.9 Hz*, 2H).

8-Bromo-2-(4-bromobenzoyl)-1H,2H-naphtho[2,1-b]pyran-3-one (14h)

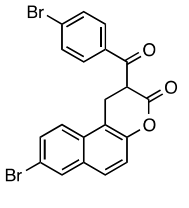

290 mg (0.63 mmol, 86%). ^{1}H NMR (300 MHz, DMSOd$_6$) δ 8.27 (d, *J = 2.1 Hz*, 1H), 8.04 (d, *J = 9.0 Hz*, 2H), 7.96 (dd, *J = 12.0, 9.0Hz*, 2H), 7.78 (d, *J = 8.4 Hz*, 2H), 7.70 (dd, *J = 9.0, 2.1 Hz*,

1H), 7.22 (d, *J = 9.0 Hz*, 1H), 5.34 (dd, *J= 11.1, 6.9 Hz*, 1H), 3.67 (ddd, *J = 16.5, 11.1, 6.9 Hz*, 2H).

8-Bromo-2-(4-methoxybenzoyl)-1H,2H-naphtho[2,1-b]pyran-3-one (14i)

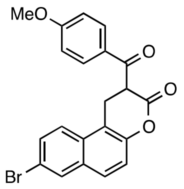

535 mg (1.31 mmol, 81%). ^{1}H NMR (300 MHz, DMSOd$_6$) δ 8.27 (d, *J = 2.1 Hz*, 1H), 8.08 (d, *J = 9.0 Hz*, 2H), 8.00-7.89 (m, 2H), 7.68 (dd, *J = 9.0, 1.8 Hz*, 1H), 7.41 (d, *J = 9.0,* 1H), 7.07 (d, *J = 9.0 Hz*, 2H), 5.31 (dd, *J= 10.2, 6.9 Hz*, 1H), 3.66 (ddd, *J = 16.8, 10.2, 6.9 Hz*, 2H), 3.87 (s, 3H).

8-Bromo-2-(4-fluorobenzoyl)-1H,2H-naphtho[2,1-b]pyran-3-one (14j)

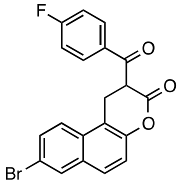

350 mg (0.81 mmol, 91%). ^{1}H NMR (300 MHz, DMSOd$_6$) δ 8.27 (d, *J = 1.8 Hz*, 1H), 8.25-8.14 (m, 2H), 7.96 (dd, *J = 11.7, 9.3 Hz*, 2H), 7.70 (dd, *J = 8.7, 1.8 Hz,* 1H), 7.48-7.34 (m, 3H), 5.36 (dd, *J= 10.8, 6.9 Hz*, 1H), 3.68 (ddd, *J = 16.5, 10.8, 6.9 Hz*, 2H).

8-bromo-2-(pyridine-4-carbonyl)-1H,2H-naphtho[2,1-b]pyran-3-one (14k)

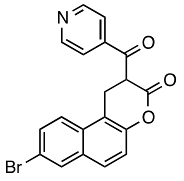

217 mg (0.57 mmol, 83%). LRMS [ES]$^-$: m/z = 380.0 (M - H)$^-$.

8-bromo-2-(pyridine-3-carbonyl)-1H,2H-naphtho[2,1-b]pyran-3-one (14l)

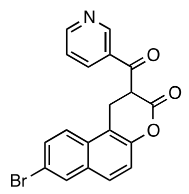

171mg (0.45 mmol, 74%).LRMS $[ES]^+$: m/z = 382.0 $(M+H)^+$.

N-(4-{8-bromo-3-oxo-1H,2H-naphtho[2,1-b]pyran-2-carbonyl}phenyl)acetamide(14m)

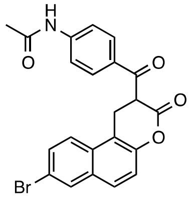

114 mg (0.26 mmol, 81%). LRMS: m/z 436.0 $(M-H)^-$.

2-(3-bromobenzoyl)-1H,2H-naphtho[2,1-b]pyran-3-one (14n)

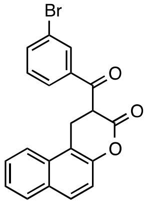

0.30 mg (0.79 mmol, 100%). ^{1}H NMR (300 MHz, $DMSOd_6$) δ 8.32 (t, *J = 2.1 Hz*, 1H), 8.10 (d, *J = 7.8 Hz*, 1H), 8.05-7.86 (m, 4H), 7.66-7.49 (m, 3H), 7.36 (d, *J = 9.0 Hz*, 1H), 5.38 (dd, *J= 11.4, 6.6 Hz*, 1H), 3.68 (ddd, *J = 16.5, 11.4, 6.6 Hz*, 2H).

8-methoxy 2-(3-bromobenzoyl)-1H,2H-naphtho[2,1-b]pyran-3-one (14o)

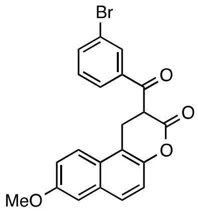

73 mg (0.18 mmol, 85%). ^{1}H NMR (300 MHz, $DMSOd_6$) δ 8.30 (s, 1H), 8.09 (d, *J = 7.8 Hz*, 1H), 7.99-7.79 (m, 3H), 7.53 (t, *J = 7.8 Hz*, 1H), 7.41 (d, *J = 2.4 Hz*, 1H), 7.31 (d, *J = 8.7 Hz*, 1H),

7.25 (dd, *J= 9.3, 2.4 Hz*, 1H), 5.35 (dd, *J= 11.4, 6.6 Hz*, 1H), 3.88 (s, 3H), 3.63 (ddd, *J = 16.5, 11.4, 6.6 Hz*, 2H).

5-bromo-2-(3-bromobenzoyl)-1H,2H-naphtho[2,1-b]pyran-3-one (14p)

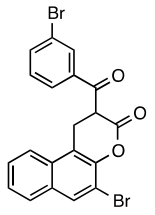

222 mg (0.48 mmol, 88%). ^{1}H NMR (300 MHz, DMSOd$_6$) δ 8.32 (bt, *J = 1.5 Hz*, 1H), 8.23 (d, *J = 1.2 Hz*, 1H), 8.10 (d, *J = 8.1 Hz*, 1H), 8.04-7.86 (m, 3H), 7.65 (dd, *J = 9.0, 1.5 Hz*, 1H),), 7.54 (t, *J = 8.1 Hz*, 1H), 7.41 (d, *J = 9.0 Hz*, 1H), 5.36 (dd, *J= 11.7, 6.6 Hz*, 1H), 3.81 (ddd, *J = 16.5, 11.7, 6.6 Hz*, 2H). LRMS [ES]$^+$: m/z = 456.9 (M-H)$^-$.

9-bromo-2-(3-bromobenzoyl)-1H,2H-naphtho[2,1-b]pyran-3-one (14q)

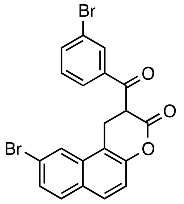

218 mg (0.48 mmol, 87%). ^{1}H NMR (300 MHz, DMSOd$_6$) δ 8.35 (s, 1H), 8.33 (t, *J = 1.5 Hz*, 1H), 8.11 (d, *J = 8.1 Hz*, 1H), 8.03 (d, *J = 8.4 Hz*, 1H), 7.97 (d, *J = 7.5 Hz*, 1H),), 7.91 (td, *J = 8.1, 1.2 Hz*, 1H), 7.67-7.60 (m, 1H), 7.60-7.51 (m, 2H), 5.41 (d, *J = 12.0, 6.6 Hz*, 1H), 3.80 (ddd, *J = 16.6, 12.0, 6.6 Hz*, 2H). LRMS [ES]$^+$: m/z = 456.9 (M-H)$^+$.

8-methyl-2-(3-trifluoromethylbenzoyl)-1H,2H-naphtho[2,1-b]pyran-3-one (14r)

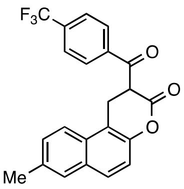

263 mg (0.68 mmol, 87%). ^{1}H NMR (300 MHz, DMSOd$_6$) δ 8.30 (d, *J = 8.1 Hz*, 2H), 8.00-7.80 (m, 4H), 7.75 (s, 1H), 7.45 (d, *J = 8.7 Hz*, 1H), 7.32 (d, *J = 9.0 Hz*, 1H), 5.41 (dd, *J= 11.1, 6.9 Hz*, 1H), 3.68 (ddd, *J = 16.5, 11.1, 6.9 Hz*, 2H), 2.47 (s, 3H). LRMS [ES]$^+$: m/z = 383.1 (M+H)$^+$.

8-chloro-2-[4-(trifluoromethyl)benzoyl]chromeno[6,5-b]pyridin-3-one (14s)

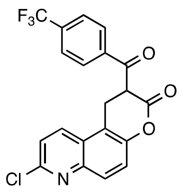

To a solution of substituted *6-Bromo-2-hydroxynaphthalene-1-carbaldehyde* (229 mg, 0.93 mmol) in ethanol (9 mL) were added the corresponding ethyl-(4-trifluoromethylphenyl)-3-oxopropanoate 193 mg (0.93 mmol). Piperidine (5 drops) was added, and the reaction was heated under reflux for 2 h. The reaction was allowed to cool, and the fine yellowish precipitate obtained was collected by filtration and washed with ethanol several times to get the condensation product 8-chloro-2-[3-(trifluoromethyl)benzoyl]-1H,2H-pyrano[3,2-f]quinolin-3-one, 89.0 mg (0.22 mmol, 24%). The reaction mixture was carried to the next step without further purification.

Quinolinone (0.89 g, 0.22 mmol) was dissolved in dry pyridine (1 mL). To this solution was added $NaBH_4$ (1.25 equiv., 0.27 mmol, 10.4 mg), and the reaction was stirred at room temp for 3 h. The mixture was then poured in cold 2 M hydrochloric acid (5 mL), which resulted in a white precipitate. The precipitate was washed several times with water, dried under a vacuum to yield 90.0 mg of (0.22 mmol, 100%) the corresponding *8-chloro-2-[4-(trifluoromethyl)benzoyl]chromeno[6,5-b]pyridin-3-one)*. 90.0 mg (0.22 mmol, 100%). ^{1}H NMR (300 MHz, DMSOd$_6$) δ 8.58 (d, *J = 9.0 Hz*, 1H), 8.37-8.25 (m, 2H), 8.02-7.91 (m, 2H), 7.67 (dd, *J = 9.0, 5.4 Hz*, 2H), 7.32 (dd, *J = 21.6, 7.32 Hz*, 1H), 5.45 (dd, *J= 11.7, 6.9 Hz*, 1H), 3.74 (ddd, *J = 16.8, 11.7, 6.9 Hz*, 2H).

Reference:

1. Mahajan, S. S.; Scian, M.; Sripathy, S.; Posakony, J.; Lao, U.; Loe, T. K.; Leko, V.; Thalhofer, A.; Schuler, A. D.; Bedalov, A.; Simon, J. A. Development of pyrazolone and isoxazol-5-one cambinol analogues as sirtuin inhibitors. *J Med Chem* **2014,** 57, 3283-94.
